# Usefulness of Examining Various Patient-Reported Outcomes in Predicting Endoscopic Mucosal Healing in Ulcerative Colitis

**DOI:** 10.5152/tjg.2022.21375

**Published:** 2022-08-01

**Authors:** Jung Min Moon, Hyuk Yoon, Jihye Park, Cheol Min Shin, Young Soo Park, Nayoung Kim, Dong Ho Lee, Joo Sung Kim

**Affiliations:** 1 Department of Internal Medicine, Liver Research Institute, Seoul National University College of Medicine, Seoul, Korea; 2 Department of Internal Medicine, Chung-Ang University College of Medicine, Seoul, Korea; 3 Department of Internal Medicine, Seoul National University Bundang Hospital, Seongnam-si, Korea; 4 Department of Internal Medicine, Institute of Gastroenterology, Yonsei University College of Medicine, Seoul, Korea

**Keywords:** Endoscopic mucosal healing, patient-reported outcomes, UCEIS, ulcerative colitis

## Abstract

**Background:**

Endoscopy remains the gold standard for evaluating mucosal healing in ulcerative colitis. However, given its invasiveness and high cost, it is not always possible to perform it as often. This study aimed to evaluate value of numerous patient-reported symptoms in the prediction of endoscopic mucosal healing.

**Methods:**

We prospectively conducted a cohort involving 143 patients with ulcerative colitis (men: 63.6%, median age: 40.0 years) in a tertiary teaching hospital between May 2017 and May 2020. Clinical remission was defined as resolution of rectal bleeding and normalization of stool frequency, set as basic patient-reported outcomes. The presence of additional 4 patient-reported outcomes (urgency, tenesmus, mucoid stool, and night defecation) were evaluated. Endoscopic activity was graded using the Ulcerative Colitis Endoscopic Index of Severity and endoscopic mucosal healing was defined as Ulcerative Colitis Endoscopic Index of Severity 0-1.

**Results:**

A total of 44 (30.77%) ulcerative colitis patients were categorized as achieving endoscopic mucosal healing. Across different patient-reported outcomes status in predicting endoscopic mucosal healing, clinical remission status inferred from basic patient-reported outcomes was superior to additional 4 patient-reported outcomes collectively (sensitivity/specificity: Ulcerative Colitis Endoscopic Index of Severity = 0/1, basic patient-reported outcomes 59.09%/75.76%, additional 4 patient-reported outcomes 70.45%/72.73%). Combination of basic and additional patient-reported outcomes revealed increased specificity of 83.84%. Multivariate analysis adjusted for age, sex, disease extent, and disease duration also revealed consistent results that patient-reported outcomes were independently associated to endoscopic mucosal healing (*P* < .001).

**Conclusion:**

Recognizing the presence of additional patient-reported outcomes may be useful in clinical practice as it is a simple and easy method that not only reflects patient’s quality of life but can also relatively better predict endoscopic mucosal healing status than basic patient-reported outcomes.

**Main Points**

- Although endoscopic evaluation in mucosal healing is an important treatment target, it is not always possible to perform it as often due to cost and invasiveness.
- In ulcerative colitis patients, extra symptoms (urgency, mucoid stool, tenesmus, and night defecation) on top of the conventional patient-reported outcomes (PROs) may be helpful in predicting endoscopic mucosal healing status.
- Therefore, simple evaluation of a few extra PROs may be beneficial in clinical decision on endoscopic evaluation interval.

## Introduction

Ulcerative colitis (UC) has been increasing in prevalence worldwide, including Asian countries.^1,2, 3^ Among existing modalities, endoscopy is considered the gold standard to assess disease activity in UC. Evaluating mucosal healing is especially crucial, as it can be considered the ultimate therapeutic endpoint.^4^ Indeed, previous studies have demonstrated the pivotal role of mucosal healing in decreasing the risk of treatment escalation, disease relapse, and colectomy.^5,6^ However, endoscopy is not always welcomed by most patients because of its high cost, cumbersome preparation, invasiveness, and possible complications.^7^

Patient-reported outcomes (PROs), rectal bleeding and stool frequency in particular, are widely used in a simple and straightforward manner to evaluate UC disease activity. In Selecting Therapeutic Targets in Inflammatory Bowel Disease (STRIDE) guidelines, clinical remission was defined as resolution of rectal bleeding and normalization of bowel habits.^4^ However, clinicians often face a dilemma as resolution of these clinical symptoms do not always correlate with endoscopic remission.^8^ A significant number of inflammatory bowel disease (IBD) patients may also have irritable bowel syndrome (IBS).^9^ Therefore, physicians often encounter difficult clinical decision-making in differentiating the “real” PROs that are truly related to the current disease activity, reflected as endoscopic mucosal healing (EMH) status. These symptomatic IBD patients may be partly due to the residual persistence of histologic inflammation.^10^

Moreover, the importance of PROs are further weighted when confronting patients’ quality of life.^9,11^ As often the disease strikes the young, they not only suffer from loss of economic productivity but also complain of other psychological disabilities such as depression and anxiety.^12^ The number of symptoms the patient suffer from may be an indicator of disease activity as well as quality of life status. Simple clinical colitis activity index (SCCAI) involves various PROs such as urgency, night defecation, and more PROs in calculation.^13^ Also, tenesmus and mucus in stool are 2 frequently reported symptoms in active UC patients.^14^

Therefore, we conducted a prospective cohort study to evaluate the value of extra PROs in addition to currently well-acknowledged PROs of rectal bleeding and stool frequency—widely used in defining clinical remission—in correlation with EMH.

## Materials and Methods

All procedures performed involving human participants were in accordance with the ethical standards of the institutional and/or national research committee and with the 1964 Helsinki declaration and its later amendments or comparable ethical standards. The study was approved by the Institutional Review Board in this teaching hospital (IRB number: B-1808-484-127). All personal data were anonymized.

### Study Population

This prospective cohort study was performed at a tertiary referral center, between May 2017 and May 2020. Patients above 18 years old with previously diagnosed UC were enrolled. This diagnosis was made when certain criteria were met with confirmation by an IBD specialist doctor. Clinical manifestation, endoscopic or radiologic findings, and pathological findings were needed to be diagnosed and registered as rare and intractable diseases in the Korean national health insurance.^15^ Only those with colonoscopy or sigmoidoscopy within 1 month to the clinic visit were included in the analysis. Patients with unclassified IBD and patients with unavailable fecal calprotectin (FCP) data within 1 week from endoscopy were excluded. Finally, 143 patients from the prospectively collected cohort were analyzed.

### Measurement of Endoscopic Activity

Either sigmoidoscopy or colonoscopy was done under clinical indications. Endoscopic activity was graded using the Ulcerative Colitis Endoscopic Index of Severity (UCEIS). Endoscopy was performed by experienced gastroenterologists specializing in IBD (HY and JP) at SNUBH. Endoscopic mucosal healing was defined as UCEIS 0-1.^16^ The UCEIS was used instead of the Mayo clinic endoscopic subscore, as the former is believed to be the only validated endoscopic index in UC with less interpersonal and intrapersonal variation.^17^

### Evaluation of Clinical Activity

Clinical information was gathered at the clinic visit of the initial patient enrollment, within a month prior to endoscopy performance. Patient-reported outcomes collected were basic PROs of rectal bleeding and stool frequency and 4 additional PROs of urgency, tenesmus, mucoid stool, and night defecation. These PROs symptoms were described in binary form: absence or presence. Clinical remission was defined as resolution of rectal bleeding and almost normalization of stool frequency as indicated in STRIDE guideline.^4^

### Fecal Calprotectin Levels

Fecal samples, requiring less than 100 mg of feces, were collected within 1 week of endoscopy. The patient brought the stool sample on the date of endoscopy or laboratory analysis. Fecal calprotectin was measured using the Quantum Blue Calprotectin rapid test (Bühlmann Laboratories, Basel, Switzerland), and laboratory personnel were blinded to the results of the colonoscopy and clinical information of the patient.

### Statistical Analysis

Continuous variables were expressed as means and standard deviations or median and interquartile ranges as to know whether the population is normally distributed; categorical variables were reported as numbers and percentages. Continuous variables were compared using the *t*-test or Mann–Whitney *U* test and categorical variables were compared using the chi-square test. To evaluate specificity, sensitivity in predicting EMH was calculated for each PRO and also for composite of basic and additional PROs, respectively. Sensitivity of PRO score was defined as the proportion of patients free of PROs when EMH was present according to endoscopy. Specificity of PRO score was defined as the proportion of patients with PRO symptoms when EMH was not present according to endoscopy. Logistic regression was performed to assess the odds ratio of each PROs in predicting EMH. Age, sex, disease extent, and disease duration were adjusted in the multivariate analysis model. R software (version 3.5.1, R foundation for Statistical Computing, Austria) was used for all data management and statistical calculations and *P* < .05 was used as a cutoff for level of significance.

## Results

Overall, 143 patients with UC were analyzed, of which 63.6% (91) were men with the median age of 40.0 years upon enrollment. Detailed baseline characteristics of the included patients are outlined in Table 1. Approximately 15% of the patients were treated with biologics at the time of study enrollment. Less than one-third of the patients had limited extent to proctitis (n = 39, 27.3%) according to Montreal classification.^18^ The most frequently complained PRO was increase in stool frequency (n = 76, 53.1%) and the second most was rectal bleeding (n = 65, 45.5%). Among the additional PROs, urgency was the most frequently observed symptom (n = 60, 42.0%). The median level of FCP was 361.9 (range: 0-2000).

### Comparison of Various PROs Between Patients With and Without EMH

By defining EMH as UCEIS 0-1, the patients were divided into 2 groups: UCEIS 2-8 group (n = 99, 69.2%) and UCEIS 0-1 group (n = 44, 30.8%). Clinical remission (CR) was set as stool frequency normalization and resolution of rectal bleeding. Clinical remission was more frequently observed in the EMH group than those without EMH (59.1% vs 24.2%, *P* < .001). With regard to additional PROs, they were all significantly less observed in the EMH patients. Of note, most of the patients with EMH were asymptomatic in the night defecation criteria (n = 42, 95.5%). More patients free of additional PROs had achieved EMH (70.5% vs 27.3%, *P* < .001). Only 20 subjects (45.5%) were asymptomatic in all PROs collected (Table 2). Upon subgroup analysis, those with disease duration of more than 3 years and achieved EMH had noticeably less symptom of rectal bleeding (<5%) as compared to other PROs. As seen, CR of 40.9% was observed in EMH and subgroup analysis of longer disease duration revealed CR of 28%, while other additional PROs seemed similar between groups (Supplementary Table 1).

### Basic and Additional PROs in Predicting Endoscopic Mucosal Healing

As shown in Table 3, CR defined by 2 basic PROs combined revealed specificity of 75.76% in predicting EMH. Single additional PRO had relatively low specificity values, but composite of all 4 additional PROs had comparable specificity of 72.73%. Combination of all basic and additional PROs demonstrated higher level of specificity in predicting EMH, which was comparably higher than FCP (83.84% vs 79.59%). Higher level of specificity would point to greater clinical value in predicting EMH.

### Sensitivity Analysis

Sensitivity analysis was performed using the definition of UCEIS 0 alone as EMH. The results are presented in Supplementary Table 2, revealing results consistent to Supplementary Table 3. Of note, upon sensitivity analysis, specificity was numerically even higher, pointing out greater significance in predicting EMH using PROs in stricter definition of UCEIS 0.

## Discussion

Although it is more practical and straightforward in reality as a clinical symptom scoring system, currently widely used partial mayo score has its share of pitfalls because of its subjective nature. Indeed, physician global assessment subscore may depend largely on patients’ symptoms. In this context, we aimed to further investigate on the clinical value of other symptoms in addition to basic PROs of the partial mayo score in predicting EMH.

Building on the basic PROs, 4 additional symptoms were collected—urgency, tenesmus, mucoid stool, and night defecation. Urgency and night frequency are parameters certified in the simple clinical colitis activity index (SCCAI).^13^ Incomplete evacuation and tenesmus are frequently noted symptoms in UC patients and need particular attention as they are closely related to patients’ quality of life.^14^ On the other hand, mucoid stool and sense of incomplete evacuation are also reported to be frequent complaints of patients with IBS.^19^ With consistency to other previous studies, our results exhibited that some patients were still symptomatic while achieving EMH.^10^ Whether it is IBS-related symptom or a hint to quiescent inflammation may be rather challenging. However, our study revealed that number of PROs may be an indicator as specificity of predicting EMH increased with additional PROs. Quick assessment of the presence of additional PROs rather than physician global assessment subscore may provide a simple hint in indirectly estimating the patient’s endoscopic status.

Acknowledging that all of the PROs were independently associated with EMH, the number of symptoms may be an indicator of EMH. Our results supported this idea as adding 4 PROs to conventional PROs of the partial mayo score improved the specificity of EMH prediction. As we were aiming for means to predict EMH and possibly reduce unnecessary endoscopies, higher specificity over sensitivity is more of a concern. Thus, simply asking for the presence of additional 4 symptoms may be helpful in deciding on the interval of endoscopic evaluation in a CR status patients. Identifying this group of patients who may not require invasive endoscopy is probably much more convenient and less costly. Focusing on specificity alone, its diagnostic value was not inferior to FCP.

These symptomatic patients with EMH may infer quiescent inflammation. Indeed literatures documented approximately between 50% and 60% patients with EMH spontaneously achieving histologic remission,^20^ and more evidence is mounting on the value of histologic remission as it is related to higher remission maintenance rate and improved clinical outcome.^21,22^ Therefore, symptomatic patients in EMH may indicate those with EMH but without deep remission.

In further analysis, we endeavored to seek if certain PROs were more particularly observed in those with longer durations. Of patients with disease duration >3 years, we noted that symptoms of stool frequency, urgency, and tenesmus were consistently frequently observed even with EMH achievement, while other symptoms such as rectal bleeding, mucoid stool, and night defecation were less frequently observed. This may add nuance that those with longer disease duration are more likely to have IBS-type symptoms combined or have intestinal sequelae. Especially considering recent data that even those achieving deep remission may complain of IBS-type symptoms suggest that more than quiescent inflammation may be needed to explain this.^9^ Chronic long-standing inflammation of UC may result in anatomical dystrophy including muscosal or mucular alterations in the gut.^23^ This may lead to motility or anorectal dysfunction without inflammation.^24^ This may explain symptoms of urgency, tenesmus, and stool frequency. Another possible theory is that damaged enteric nervous system due to chronic inflammation may cause motility dysfunction.^25^ Microbiota alteration may also affect enteric nervous system as well as serotonin production via enterocromaffin cells in the gut mucosa.^26^ Future studies are needed to investigate on the functional analysis beyond occult inflammation.

To note, clinical symptoms such as mucus in stool may overlap with symptoms of IBS^27^; they should be interpreted with caution in UC patients. A meta-analysis shows prevalence of more than 30% of IBS and/or post-inflammatory IBS in IBD patients.^28^ In real world, differentiating true IBD symptoms from IBS symptoms is a particularly important but challenging issue, as management greatly differs and directly corresponds to patient’s general well-being. Varying ability of patients to differentiate mucus in stool from normal stool, because of medications such as 5-aminosalisilic acid (5-ASA) compounds, makes it even more difficult. Most oral 5-ASA agents are absorbed poorly in the systemic circulation and remain in the terminal ileum to the colon lumen. Consequently, sometimes these 5-ASA granules may be excreted in the feces, which can be mistaken as mucus in stool. In fact, among patients diagnosed with EMH, 5-aminosalicylates were prescribed to those complaining of mucus in stool. Thus, the above results imply that patients experiencing 2 or more clinically suspicious symptoms must be cautiously considered for possible co-existence of IBS-type symptoms. These patients with coincident IBS must not be underestimated as it is reported to be prevalent (35-40%),^9^ and it may also severely undermine patients’ quality of life as often directed to disability issue.^11,29^

Moreover, scrutinizing the data we found that more than one-third of the subpopulation without any PROs were in fact endoscopically active (6/36, 44.4%). They were shorter in disease duration (21.3 vs 57.9) and revealed much higher level of FC (461.6 vs 82.0) compared to those with EMH. It may be inferred that those with relatively shorter disease duration should therefore be dealt with particular caution.

This study had some limitations to acknowledge. This study has a relatively small sample size. In addition, clinical symptoms additionally gathered were binomial data. Accordingly, minute symptoms that were clinically insignificant may have been classified as the presence of symptoms. However, bearing in mind that the primary outcome was to focus on complete EMH and these patients presented with few symptoms, our data could be successfully analyzed in dichotomous manner. Nevertheless, follow-up study may be enriched by subdividing the symptom score into 3 point scale data, comparable to partial mayo score. Further gathering of patient quality of life data via questionnaire may provide in-depth information. Moreover, some portion of sigmoidosopcy alone data was also included in the analysis, which may partly effect on scoring endoscopic activity. However, previous literatures have confirmed a significant concordance of colonoscopy and sigmoidoscopy in the evaluation of UC.^30,31^ Acknowledging that not every patient with acute symptoms is qualified for bowel preparation and full colonoscopy, this population may rather represent real-life practice. Lastly, future study on the longitudinal follow-up data including histologic remission and relapse free status could clarify on the association between remnant microscopic inflammations and PROs.

In conclusion, adding extra symptoms, such as urgency, mucoid stool, tenesmus, and night defecation, in addition to the conventional PROs of rectal bleeding and stool frequency may be beneficial in clinical decision on endoscopic evaluation interval. However, some patients with longer disease duration may present with various symptoms even with EMH, which may possibly point to co-incident IBS-type symptoms. As these may seriously compromise one’s quality of life, it should be interpreted meticulously with careful approach in UC patients.

## Figures and Tables

**Table 1.** Baseline Characteristics of the Study Population

Characteristics	Value
No. of patients	143
Age, years	40.0 (31.5-54.0)
Male	91 (63.6%)
Disease duration, months	32.0 (10.5-68.3)
Colonoscopy	101 (70.6%)
Extent	
Proctitis	39 (27.3%)
Left-side colitis	51 (35.7%)
Extensive colitis	53 (37.1%)
Concomitant medications	
Aminosalicylate	116 (81.12%)
Azathiopurine/mercaptopurine	19 (13.29%)
Corticosteroids	23 (16.08%)
Biologics	22 (15.38%)
Basic patient-reported outcomes	
Stool frequency	
0	67 (46.9%)
1	76 (53.1%)
Rectal bleeding	
0	78 (54.5%)
1	65 (45.5%)
Additional patient-reported outcomes	
Urgency	60 (42.0%)
Tenesmus	54 (37.8%)
Mucoid stool	43 (30.1%)
Night defecation	31 (21.7%)
Fecal calprotectin, ug/g	361.9 (76.3-1305.2)
Fecal immunochemical test	0 (0-627.5)
hs-C-reactive protein, mg/dL	0.11 (0.04-0.36)
Ulcerative Colitis Endoscopic Index for Severity	
0	24 (16.78%)
1	20 (13.99%)
2	19 (13.29%)
3	19 (13.29%)
4	19 (13.29%)
5	22 (15.38%)
6	15 (10.49%)
7	5 (3.50%)
8	0 (0%)

Values are presented as number (%) or median (interquartile range).

**Table 2.** Basic and Additional Patient-Reported Outcomes of the Patients with Mucosal Healing Defined as Ulcerative Colitis Endoscopic Index for Severity (UCEIS) 0-1

		UCEIS 0-1 (N = 44)	UCEIS 2-8 (N = 99)	*P*
Basic PROs				
Stool frequency, n (%)	−	31 (70.5%)	36 (36.4%)	<.001
	+	13 (29.5%)	63 (63.6%)	
Rectal bleeding, n (%)	−	38 (86.4%)	40 (40.4%)	<.001
	+	6 (13.6%)	59 (59.6%)	
Additional PROs				
Urgency, n (%)	−	36 (81.8%)	47 (47.5%)	<.001
	+	8 (18.2%)	52 (52.5%)	
Tenesmus, n (%)	−	36 (81.8%)	53 (53.5%)	.002
	+	8 (18.2%)	46 (46.5%)	
Mucoid stool, n (%)	−	38 (86.4%)	62 (62.6%)	.008
	+	6 (13.6%)	37 (37.4%)	
Night defecation, n (%)	−	42 (95.5%)	70 (70.7%)	.002
	+	2 (4.5%)	29 (29.3%)	
Clinical Remission (CR)^1^	+	26 (59.1%)	24 (24.2%)	<.001
	−	18 (40.9%)	75 (75.8%)	
Additional four PROs^2^	0	31 (70.5%)	27 (27.3%)	<.001
	≥1	13 (29.5%)	72 (72.7%)	
CR + additional four PROs	0	20 (45.5%)	16 (16.2%)	<.001
	≥1	24 (54.5%)	83 (83.8%)	

^1^Clinical remission defined as stool frequency 0 and rectal bleeding 0.

^2^Additional 4 RPOs defined as summation of urgency, tenesmus, mucoid stool, and night defecation.

CR, clinical remission; PROs, patient-reported outcomes; UCEIS, Ulcerative Colitis Endoscopic Index for Severity.

**Supplementary Table 1.** Subgroup Analysis Depending on Disease Duration (Longer than 3 Years)

Patient-reported outcomes		UCEIS 0-1 (N = 25)	UCEIS 2-8 (N = 42)	*P*
Basic				
Stool frequency, n (%)	−	19 (76.0%)	13 (31.0%)	.001
	+	6 (24.0%)	29 (69.0%)	
Rectal bleeding, n (%)	−	24 (96.0%)	16 (38.1%)	<.001
	+	1 (4.0%)	26 (61.9%)	
Additional				
Urgency, n (%)	−	20 (80.0%)	18 (42.9%)	.007
	+	5 (20.0%)	24 (57.1%)	
Tenesmus, n (%)	−	22 (88.0%)	17 (40.5%)	<.001
	+	3 (12.0%)	25 (59.5%)	
Mucoid stool, n (%)	−	23 (92.0%)	23 (54.8%)	.004
	+	2 (8.0%)	19 (45.2%)	
Night defecation, n (%)	−	24 (96.0%)	30 (71.4%)	.032
	+	1 (4.0%)	12 (28.6%)	
Clinical remission^1^	+	18 (72.0%)	8 (19.0%)	<.001
	−	7 (28.0%)	34 (81.0%)	
Additional 4 PROs^2^	0	19 (76.0%)	11 (26.2%)	<.001
	≥1	6 (24.0%)	31 (73.8%)	

^1^Clinical remission defined as stool frequency 0 and rectal bleeding 0

^2^Additional 4 PROs defined as summation of urgency, tenesmus, mucoid stool, and night defecation.

**Table 3.** Prediction of Endoscopic Mucosal Healing by Basic and Additional Patient-Reported Symptoms and Laboratory Biomarkers

	UCEIS (n)		Sensitivity	Specificity
0-1	≥2
Clinical remission^1^	26	24	59.09	75.76
Additional PROs^2^				
Urgency (−)	36	47	81.82	52.53
Tenesmus (−)	36	53	81.82	46.46
Mucoid stool (−)	62	38	86.36	37.37
Night defecation (−)	42	70	95.45	29.29
Additional 4 PROs (−)	85	58	70.45	72.73
CR + urgency (−)	24	23	54.55	76.77
CR + tenesmus (−)	23	20	52.27	79.80
CR + mucoid stool (−)	25	23	56.82	76.77
CR + night defecation (−)	26	22	59.09	77.78
CR + additional PROs 0	20	16	45.45	83.84
Fecal calprotectin < 250 µg/g	33	20	75.00	79.59

^1^Clinical Remission defined as stool frequency 0 and rectal bleeding 0.

^2^Additional 4 PROs defined as summation of urgency, tenesmus, mucoid stool, and night defecation.

CR, clinical remission; PROs, patient-reported outcomes.

**Supplementary Table 2.** Prediction of UCEIS 0 by Basic and Additional Patient-Reported Symptoms and Laboratory Biomarkers

	UCEIS (n)		Sensitivity	Specificity
0	≥1
Clinical remission (CR)^1^	15	35	62.50	70.59
Additional PROs^2^				
Urgency (−)	20	63	83.33	47.06
Tenesmus (−)	21	68	87.50	42.86
Mucoid stool (-)	23	89	91.67	34.45
Night defecation (−)	22	78	95.83	20.21
Additional 4 PROs (−)	17	41	70.83	65.55
CR + urgency (−)	13	34	54.17	71.43
CR + tenesmus (−)	13	30	54.17	74.79
CR + mucoid stool (−)	15	33	58.33	71.43
CR + night defecation (−)	14	34	62.50	72.27
CR + additional PROs 0	10	26	41.67	78.15
Fecal calprotectin < 250 µg/g	19	45	79.17	61.86

^1^Clinical remission defined as stool frequency 0 and rectal bleeding 0.

^2^Additional 4 PROs defined as summation of urgency, tenesmus, mucoid stool, and night defecation.

PROs, patient-reported outcomes.

**Supplementary Table 3.** Comparison of Those with Sustained Endoscopic Activity and Endoscopic Remission Among Patients Without Any PROs

Characteristics	Endoscopic Active (N = 16)	Endoscopic Remission (N = 20)	*P*
Age, years	39.6 ± 12.4	41.9 ± 15.5	.627
Male	11 (68.8%)	11 (55.0%)	.619
Disease duration, months	21.3 (7.9-47.2)	57.9 (30.6-90.6)	.052
Fecal calprotectin	461.6 (170.9-1179.5)	82.0 (27.1-212.2)	.005
Extent			.932
Proctitis	5 (31.2%)	7 (35.0%)	
Left-side colitis	4 (25.0%)	4 (20.0%)	
Extensive colitis	7 (43.8%)	9 (45.0%)	

## References

[1] Kaplan GG Ng SC . Understanding and preventing the global increase of inflammatory bowel disease. Gastroenterology. 2017;152(2):313 321.e2. 10.1053/j.gastro.2016.10.020) 27793607

[2] Park SH Kim YJ Rhee KH et al. A 30-year Trend Analysis in the Epidemiology of Inflammatory Bowel Disease in the Songpa-Kangdong District of Seoul, Korea in 1986-2015. J Crohns Colitis. 2019;13 :1410 1417. 10.5217/ir.2018.00096) 30989166

[3] Yen HH Weng MT Tung CC et al. Epidemiological trend in inflammatory bowel disease in Taiwan from 2001 to 2015: a nationwide populationbased study. Intest Res. 2019;17(1):54 62. 10.5217/ir.2018.00096) 30449079 PMC6361021

[4] Peyrin-Biroulet L Sandborn W Sands BE et al. Selecting therapeutic targets in inflammatory bowel disease (STRIDE): determining therapeutic goals for treat-to-target. Am J Gastroenterol. 2015;110(9):1324 1338. 10.1038/ajg.2015.233) 26303131

[5] Ardizzone S Cassinotti A Duca P et al. Mucosal healing predicts late outcomes after the first course of corticosteroids for newly diagnosed ulcerative colitis. Clin Gastroenterol Hepatol. 2011;9(6):483 .e3 489.e3. 10.1016/j.cgh.2010.12.028) 21195796

[6] Neurath MF Travis SP . Mucosal healing in inflammatory bowel diseases: a systematic review. Gut. 2012;61(11):1619 1635. 10.1136/gutjnl-2012-302830) 22842618

[7] Higgins PD Schwartz M Mapili J Zimmermann EM . Is endoscopy necessary for the measurement of disease activity in ulcerative colitis? Am J Gastroenterol. 2005;100(2):355 361. 10.1111/j.1572-0241.2005.40641.x) 15667493

[8] Jharap B Sandborn WJ Reinisch W et al. Randomised clinical study: discrepancies between patient-reported outcomes and endoscopic appearance in moderate to severe ulcerative colitis. Aliment Pharmacol Ther. 2015;42(9):1082 1092. 10.1111/apt.13387) 26381802 PMC5049645

[9] Fairbrass KM Costantino SJ Gracie DJ Ford AC . Prevalence of irritable bowel syndrome-type symptoms in patients with inflammatory bowel disease in remission: a systematic review and meta-analysis. Lancet Gastroenterol Hepatol. 2020;5(12):1053 1062. 10.1016/S2468-1253(20)30300-9) 33010814

[10] Colombel JF Keir ME Scherl A et al. Discrepancies between patient-reported outcomes, and endoscopic and histological appearance in UC. Gut. 2017;66(12):2063 2068. 10.1136/gutjnl-2016-312307) 27590995 PMC5749342

[11] Gracie DJ Williams CJ Sood R et al. Negative effects on psychological health and quality of life of genuine irritable bowel syndrome-type symptoms in patients With inflammatory bowel disease. Clin Gastroenterol Hepatol. 2017;15(3):376 384.e5. 10.1016/j.cgh.2016.05.012) 27189912

[12] Longobardi T Jacobs P Bernstein CN . Work losses related to inflammatory bowel disease in the United States: results from the National Health Interview Survey. Am J Gastroenterol. 2003;98(5):1064 1072. 10.1111/j.1572-0241.2003.07285.x) 12809829

[13] Walmsley RS Ayres RC Pounder RE Allan RN . A simple clinical colitis activity index. Gut. 1998;43(1):29 32. 10.1136/gut.43.1.29) 9771402 PMC1727189

[14] Rao SS Holdsworth CD Read NW . Symptoms and stool patterns in patients with ulcerative colitis. Gut. 1988;29(3):342 345. 10.1136/gut.29.3.342) 3356365 PMC1433596

[15] Moon JM Kang EA Han K et al. Trends and risk factors of elderly-onset Crohn’s disease: a nationwide cohort study. World J Gastroenterol. 2020;26(4):404 415. 10.3748/wjg.v26.i4.404) 32063689 PMC7002904

[16] Vuitton L Peyrin-Biroulet L Colombel JF et al. Defining endoscopic response and remission in ulcerative colitis clinical trials: an international consensus. Aliment Pharmacol Ther. 2017;45(6):801 813. 10.1111/apt.13948) 28112419

[17] Travis SP Schnell D Krzeski P et al. Reliability and initial validation of the ulcerative colitis endoscopic index of severity. Gastroenterology. 2013;145(5):987 995. 10.1053/j.gastro.2013.07.024) 23891974

[18] Satsangi J Silverberg MS Vermeire S Colombel JF . The Montreal classification of inflammatory bowel disease: controversies, consensus, and implications. Gut. 2006;55(6):749 753. 10.1136/gut.2005.082909) 16698746 PMC1856208

[19] Manning AP Thompson WG Heaton KW Morris AF . Towards positive diagnosis of the irritable bowel. Br Med J. 1978;2(6138):653 654. 10.1136/bmj.2.6138.653) 698649 PMC1607467

[20] Kanazawa M Takahashi F Tominaga K et al. Relationship between endoscopic mucosal healing and histologic inflammation during remission maintenance phase in ulcerative colitis: a retrospective study. Endosc Int Open. 2019;7(4):E568 E575. 10.1055/a-0869-7619) 30957007 PMC6449158

[21] Christensen B Hanauer SB Erlich J et al. Histologic normalization occurs in ulcerative colitis and is associated With improved clinical outcomes. Clin Gastroenterol Hepatol. 2017;15(10):1557 .e1 1564.e1. 10.1016/j.cgh.2017.02.016) 28238954 PMC5618439

[22] Yoon H Jangi S Dulai PS et al. Incremental benefit of achieving endoscopic and histologic remission in patients with ulcerative colitis: a systematic review and meta-analysis. Gastroenterology. 2020;159(4):1262 1275.e7. 10.1053/j.gastro.2020.06.043) 32585306 PMC7658293

[23] Torres J Billioud V Sachar DB Peyrin-Biroulet L Colombel JF . Ulcerative colitis as a progressive disease: the forgotten evidence. Inflamm Bowel Dis. 2012;18(7):1356 1363. 10.1002/ibd.22839) 22162423

[24] Latella G Di Gregorio J Flati V Rieder F Lawrance IC . Mechanisms of initiation and progression of intestinal fibrosis in IBD. Scand J Gastroenterol. 2015;50(1):53 65. 10.3109/00365521.2014.968863) 25523556

[25] Lakhan SE Kirchgessner A . Neuroinflammation in inflammatory bowel disease. J Neuroinflammation. 2010;7:37. 10.1186/1742-2094-7-37) PMC2909178 20615234

[26] Lee SH Kwon JE Cho ML . Immunological pathogenesis of inflammatory bowel disease. Intest Res. 2018;16(1):26 42. 10.5217/ir.2018.16.1.26) 29422795 PMC5797268

[27] Ford AC Bercik P Morgan DG Bolino C Pintos-Sanchez MI Moayyedi P . Validation of the Rome III criteria for the diagnosis of irritable bowel syndrome in secondary care. Gastroenterology. 2013;145(6):1262 70.e1. 10.1053/j.gastro.2013.08.048) 23994201

[28] Halpin SJ Ford AC . Prevalence of symptoms meeting criteria for irritable bowel syndrome in inflammatory bowel disease: systematic review and meta-analysis. Am J Gastroenterol. 2012;107(10):1474 1482. 10.1038/ajg.2012.260) 22929759

[29] Ye BD Travis S . Improving the quality of care for inflammatory bowel disease. Intest Res. 2019;17(1):45 53. 10.5217/ir.2018.00113) 30449081 PMC6361018

[30] Magro F Langner C Driessen A et al. European consensus on the histopathology of inflammatory bowel disease. J Crohns Colitis. 2013;7(10):827 851. 10.1016/j.crohns.2013.06.001) 23870728

[31] Colombel JF Ordás I Ullman T et al. Agreement Between rectosigmoidoscopy and colonoscopy analyses of disease activity and healing in patients With ulcerative colitis. Gastroenterology. 2016;150(2):389 .e3 39 5.e3. 10.1053/j.gastro.2015.10.016) 26526713

